# Exploring Oral Health Challenges and Barriers to Dental Care Among Children in Cabo Verde: A Qualitative Study

**DOI:** 10.1002/puh2.70184

**Published:** 2026-01-13

**Authors:** Onaedo Ilozumba, Marijke W. Visser, J. J. (Hans) de Soet, Catherine M. C. Volgenant

**Affiliations:** ^1^ Department of Applied Health Sciences School of Health Sciences University of Birmingham Birmingham UK; ^2^ Faculty of Earth and Life Sciences (FALW) Athena Institute Vrije Universiteit Amsterdam Amsterdam the Netherlands; ^3^ Department of Preventive Dentistry Academic Centre for Dentistry Amsterdam (ACTA) University of Amsterdam and Vrije Universiteit Amsterdam Amsterdam the Netherlands; ^4^ Department of Cariology Academic Centre for Dentistry Amsterdam (ACTA) University of Amsterdam and Vrije Universiteit Amsterdam Amsterdam the Netherlands

**Keywords:** dental health, oral health, prevention, small island developing states

## Abstract

**Objectives:**

Global oral health is a crucial topic since this (largely preventable) burden affects 3.5 million people worldwide, disproportionately impacting disadvantaged groups and exacerbating in low‐ and middle‐income countries like Cabo Verde. Our aim in this exploratory study was to understand the oral health landscape for children in Sal, Cabo Verde.

**Methods:**

Through a purposive sampling strategy and snowballing approach, we identified 38 stakeholders, including 20 schoolchildren and 8 parents. Data were collected through semi‐structured interviews and focus groups. All interviews were transcribed and analyzed using an inductive thematic approach.

**Results:**

Three main themes emerged from the data: oral health knowledge and practices, dietary habits, and dental service accessibility. Key challenges included time constraints limiting comprehensive oral health education in schools; parental struggles in managing children's habits; pervasive availability of sugar‐rich foods driven by affordability and social influence; provision of sweets by tourists reinforcing uncontrolled sugar intake; and limited access to professional oral health care. Community associations played a dual role, offering support but lacking structured oral health initiatives.

**Discussion:**

This study highlights systemic barriers to oral health among Cabo Verdean children and reveals unique local dynamics. Tourism—while economically beneficial—unintentionally contributes to poor oral health through sweets distribution and shifting dietary habits, a factor rarely addressed in the literature. Limited access to affordable care and underutilized community associations further exacerbate challenges. These findings call for integrated strategies that strengthen community‐based programs and embed oral health considerations into tourism and education policies.

## Graphical abstract

Our findings suggest poor oral health among children in Cabo Verde, driven by dietary patterns, including tourist‐provided sweets, limited fresh produce access, and inadequate affordable dental services. Educational interventions in schools and community groups present key opportunities for oral health improvement.

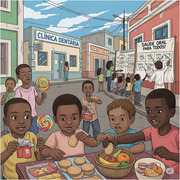

## 1 Introduction

The World Health Organization defines oral health as the state of the mouth, teeth, and orofacial structures that enable individuals to perform essential functions such as eating, breathing, and speaking. In their definition, oral health also includes a psychosocial element, including well‐being [1]. Given the widespread impact of oral health, maintaining good oral health is essential for a sufficient quality of life. Despite this and the preventable nature of oral health diseases, there is a high global burden. Most recent estimates suggest that globally, 3.5 billion people suffer from oral health problems [2].

Globally, the highest burden and prevalence of oral health problems occur in disadvantaged groups [1]. However, in low‐ and middle‐income countries (LMICs), addressing oral health problems is worsened by the existing health system constraints, including the paucity of health providers and limited access to dental treatment [3, 4]. This creates a system in which prevention of oral health issues through health promotion is preferable but also a significant challenge.

Educational institutes provide one of the most effective settings for oral health promotion and oral disease prevention [5]. Risk behaviors related to oral health are often developed during childhood, and previous studies have shown that oral health promotion and disease prevention are most effective when integrated during school‐age years [6]. Oral health education helps children to develop knowledge, skills, healthy behaviors, and positive attitudes towards oral health [7]. A recent review by Akera et al. highlighted the effectiveness of primary school‐based interventions in reducing the burden of children in LMICs [7]. Moreover, community‐based approaches to promote oral health can provide guidance and can reinforce both children and their parents [8].

However, in research‐constrained and island settings, like Cabo Verde, little is known about oral health promotion activities for children. There is research that suggests that globalization as well as changes in diet, particularly an increase in the consumption of sugary foods, has led to an increase in oral health issues [9]. In 2017, a study showed the high prevalence of oral health problems (including dental caries and severe periodontal disease [10]). In this article, we aim to fill this gap by conducting an exploratory study on the resource‐constrained island of Sal in Cabo Verde. Our study aimed to understand the oral health landscape for children in Cabo Verde. With this knowledge, we hope to provide guidance for oral health promotion and disease prevention in similar resource‐constrained settings.

## 2 Methodology

### 2.1 Study Setting

The island of Sal in Cabo Verde is a rural area consisting of one city (Espargos), a few smaller towns (of which Santa Maria is the most important), and multiple slums. The school system provides 5 hours of education per day, in the morning (Grades 1–6) or in the afternoon (Grades 7–12) [11]. During the remaining half of the day, supervised activities for children are facilitated by shelters. These shelters are widely visited by children from families of all income statuses. They are either government‐provided or administered by private organizations. Private shelters take care of both children of families that pay fees and include social support children as well. These private shelters are often sponsored by hotels and travel companies. The sole governmental shelter is open to all inhabitants and collaborates with child protection services. Next to shelters, there are associations that provide activities for the entire communities, including church kindergartens, an evening school for adults and sport associations. The oral healthcare system for the entire island comprises one dentist in the public hospital and four dentists in separate private clinics.

### 2.2 Participants

In this study, we aimed to include key stakeholders involved in the oral health of schoolchildren and their support systems. In collaboration with officials from the Sal municipality, we employed a purposive sampling strategy combined with a snowballing approach to gather relevant perspectives. A total of 38 participants were recruited: 20 schoolchildren (recruited through local shelters), 8 parents, 4 teachers of shelters (TIAs), 3 shelter directors, 1 school principal, 1 private dentist, and 1 priest. To ensure the anonymity of this small population, we do not report additional personal characteristics of the participants.

### 2.3 Data Collection

We utilized semi‐structured interviews and focus groups. MV also kept an observational logbook during her 3‐month residency at Sal to better understand the context of the study and to identify (other) underlying factors related to oral health. Interview and focus group questions were adapted for different groups, and we include an overview of sample questions in Appendix 1. Informed consent was obtained from the participants, and, for children under 18 years of age, consent was additionally secured from their caregivers. A competent local translator facilitated communication in interviews and focus group discussions (FGDs) by explaining the questions in Crioulo and translating the participants responses into English. Transcripts were developed from the English portion of the interviews, and any ambiguities in terminology or phrases were discussed with the translator.

### 2.4 Interviews

Eight face‐to‐face semi‐structured in‐depth interviews were conducted to explore the community customs and underlying factors influencing oral health [12]. An overview of the purposes of the research objectives was given to all participants, permission for recording of the conversation was asked, and a written informed consent was obtained. During the interviews, a flip‐over, pens, and sticky notes were used to visually summarize what was discussed. Interview durations ranged between 45 and 60 minutes.

### 2.5 Focus Groups

Five FGDs were conducted to understand attitudes and experiences within the community concerning oral health. Each FGD comprised 4–10 participants, with a preliminary explanation of the purpose, duration, and assurance of anonymity. In addition to the translator, MV was accompanied by a mediator who supported a confidential environment. To facilitate discussions, a flip‐over, sticky notes, and markers were used to capture and visualize key points. FGDs ranged between 60 and 90 minutes in duration.

### 2.6 Data Analysis

To maintain the exploratory nature of the study and allow for adaptation to emerging themes rather than being constrained by a pre‐established model, an inductive thematic approach was utilized. Data analysis started with MV and OI both independently reading all transcripts and developing codes. Developed codes were discussed with the entire research team of MV, OI, JJS, and CV. OI then undertook the next phase of reanalyzing all the transcripts using NVIVO13. Final themes were agreed upon in discussion between the research team. Fieldnotes were not analyzed but served as a resource for recalling specifics about interview and FGD contexts and processes.

## 3 Results

**FIGURE 1:**
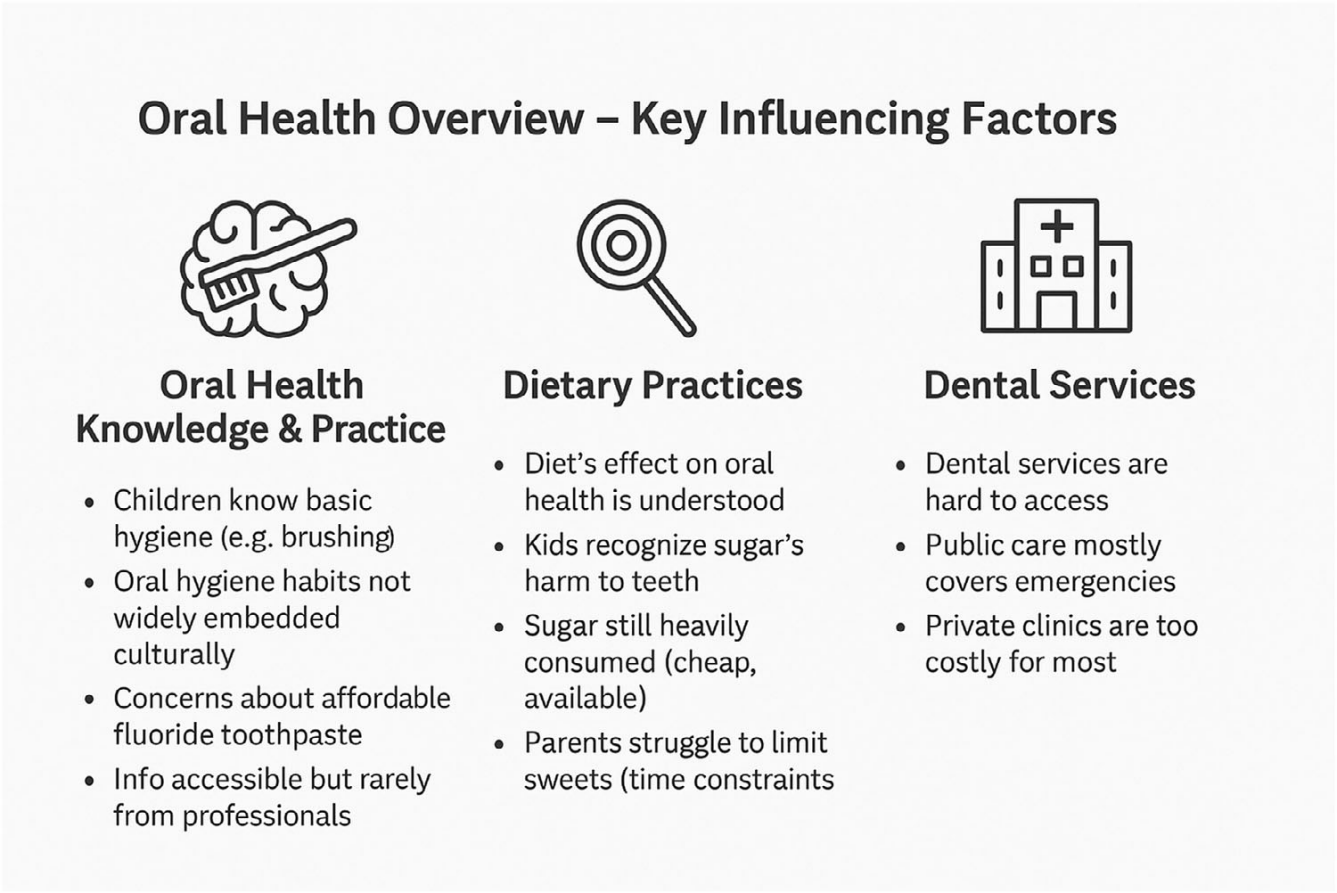
Summary of the most important issues leading to the oral health status of children on Sal, Cape Verde.

Our analysis showed that participant responses could be classified according to three main themes: oral health knowledge and practices, dietary practices, and availability and accessibility of (professional) oral health services. Figure 1 presents our key themes and findings.

### 3.1 Oral Health Knowledge and Practice

We found that school‐based interventions (donor funded), and national TV programs and the internet provided general information and education on oral health. Parents predominantly reported obtaining their information from the internet or (national) TV programs covering dental hygiene and oral diseases. Oral health education is included in the general school curriculum of the human body, and the children we spoke with demonstrated knowledge of basic oral health practices, such as brushing frequencies or the correlation between sugar consumption and tooth pain. However, the school director who was interviewed highlighted time constraints as a hindrance to providing comprehensive oral health education. The current system relies on infrequent and irregular sessions with private and international dentists for more comprehensive oral health education. Although the children received information, some participants emphasized the need for educating parents to reinforce oral hygiene habits at home.

The families, the parents. [laughs]. We can teach the kids, but if the parents don't understand how important it is to brush the teeth after lunch or dinner the kids don't do it. They don't tell them they have to do it. You can teach the children and give them a toothbrush but if they don't, the parents don't brush their teeth why do you do it? We have a solution for schools but what about the parents and the families? (Civil Servant)

Dentists were not widely recognized as a significant source of information, which the interviewed dentist attributed to parents’ lack of interest in prevention of oral diseases. This contrasted with parents expressed need for dental treatments, and instruction to specific oral hygiene and techniques, such as the origin and reduction of gum bleeding and effects of sugar consumption. They also had concerns about encouraging particularly older children to brush their teeth.

She said when this son of her was young, he used to brush the teeth, no problem. But when he was growing up, because he is 17 years old. He lost that routine. She is saying that the guy that she is talking about thinks he is already a man. He doesn't want to listen to what she says. So he can brush his teeth whenever. When he wants you know. (Mothers’ words as translated by the translator)

Participants also discussed the changes in the oral health landscape, noting that oral health products like toothpaste and toothbrushes are now available in Sal, contrasting with a prior absence. However, concerns remain regarding the availability of specialized toothpaste for children, the affordability of toothpaste for families with limited funds, and the quality of the oral health products in local shops.

The price [of the products from the local supermarket] is very low but my problem is the quality. We hear a lot of bad things about their products. They use plastic in their rice. Maybe their toothpastes and toothbrushes are from the same quality (Government shelter director)

Some parents also noted limited access to free oral health information and education through both public and private health services, although government officials asserted that dental care for children on Sal is provided at no cost and is widely recognized.

### 3.2 Dietary Practices

Dietary practices were discussed by all participants as crucial for good oral health in both children and the general population. Participants, including children, were all able to highlight that the consumption of sugar‐rich foods such as sweets, ice‐cream, cake, and sodas is detrimental to oral health. However, consumption of such foods remained high, driven by several highlighted factors.

First, all participants acknowledged the widespread accessibility of sugar‐rich foods, attributed to both the availability of these products in shops and their affordability. There were some differences in opinions about how children came to have sweets. For interviewees in managerial positions, there was a perception that parents were not concerned about their children's sweets consumption. However, other participants, including parents, discussed that parents had difficulty controlling their children's sweet consumption. This was linked to parents’ jobs which meant that they had to provide cash to their children to purchase lunch as well as the influence of others, such as family members, peers, and tourists.

She can control what she does to the kids, but not what the other person do, like on school. So it is quite difficult to control. So she said she can like tell the family, related people, that she doesn't want them to give to the kids, sweets, but it is quite difficult. (Mothers’ words as translated by the translator)

Notably, three participants specifically mentioned tourists as a source of sweets for children on the island.

Moreover, tourists give a lot of sweets to the kids on the streets. They do not understand that the children here in Sal do not have the same care as where they come from. In many cases, you have seen tourists in the supermarket buying packages of sweets. Also, for the people that live here, giving sweets is a reward. If you win, you get sweets. If you behave, you get sweets. Not only the kids have problems with their teeth. Many adults have many problems too. (Children's Centre Director)

Another factor that contributed to sweet consumption was the fact that healthier alternatives such as fruits are scarce and expensive on Sal with vegetables more commonly available. In one of the schools, hotels provide meal options for the children, but even with that, the school was only able to provide fruit on 1 day a week.

They cannot give every day…It is expensive…What the hotels give is meat and vegetables once a week for all week. Mostly vegetables. Sometimes they write letters to the business places where they sell fruits. To people who import fruits. They sent letters if they can help. Sometimes they give, sometimes they don't…They try to have what really can be shared with everybody. Like fruit is really expensive. They try to make what can be shared. (School Director)

### 3.3 Availability and Accessibility of (Professional) Oral Health Services

All participants agreed on the difficulties related to accessing dental services in Sal. The sole publicly funded dentist was situated in the public hospital in Espargos. This service was available at low cost and potentially through municipal payment aid for the unemployed. However, one participant discussed the lack of basic facilities for dental care. He gave the example of the only available dental chair being out of order, and the hospital was waiting for a new one to arrive:

They were waiting for another one, but I really don't know when. I had six months going to the hospital, asking about the dentist and they said the same thing. No chair. [And then you went to the private clinic?] Yes. I had to pay 3000 Eskudos [This is approximately 27 euros, which is 10% of a month's income]. (Priest)

Participants also deliberated on the need for more public dentists on the island. The discussion encompassed the high workload of the sole public dentist on the island and the disparity in distribution of dental services on Cabo Verde. For many participants in Sal, the only option was to seek the more expensive private dentists. However, nearly all participants mentioned the private clinics are unaffordable for the general population of Sal due to low socio‐economic status, high unemployment rates, and relatively high costs for dental treatment.

When we go to the hospital. That is a place where anyone can go, because it is a public hospital. And you wish to go there and to find a doctor, a dentist. And you go there and most of the time you will not find no one there to solve your problem. Thats the problem that we are having. A lot of children have teeth problem because we don't have a dentist. We need a dentist. And the dentists that we have here are private. And they are, you will pay a lot of money to go to the dentist. And most of the families, the poor families, with bad teeth situation, they don't have money to go to clinic. (Governmental Director)

Currently, there are discussions about community associations serving as a source of oral health care on the islands. However, these associations do not consist of dentists and are only able to provide education and support in accessing already existing services.

We have the associations of the communities. They work like a bridge. The communities and health post, the camera municipal. They help the families; give the information they need. They association is apart from the municipality. We work together. When families have a problem, we help to give solutions for their problems. They can come to the municipality; they can go to the hospital; it depends on what the family needs. So, we don't always have to be in the communities. Communities have the associations that come to us. (Civil Servant)

## 4 Discussion

This study aimed to explore the oral health landscape for children in Cabo Verde, and we found that the opportunities and barriers were both individual and systemic. Systemic barriers, particularly limited access to dental services and socio‐economic determinants, emerged as the most significant impediments to improving children's oral health in the region. This is consistent with findings in other island LMICs where healthcare infrastructure and economic constraints hamper preventive care [12, 13] and reflects a persistent global pattern in oral health inequities, where low‐resource settings bear disproportionate burdens [2, 3].

A particularly novel and important finding of this study was the role of tourism. Cabo Verde is composed of 10 islands with varying levels of development but shared economic dependence on tourism. The islands illustrate how tourism functions as a double‐edged sword [14, 15]. Although toursim contributes significantly to economic growth, the sector also influences local dietary patterns by increasing the availability and marketing of sugar‐rich products targeted at visitors [16]. As our data suggest, these changes indirectly affect children's sugar intake and oral health outcomes. Although limited research exists on the direct relationship between tourism and child oral health, our findings are consistent with broader literature on the influence of globalization and tourism on food systems in small island nations [9, 17]. Thus, tourism emerges as a highly relevant but underexplored determinant in shaping oral health risks.

Though focused on children, our study uncovered oral health challenges extending across the general population. Recent studies have shown that low health literacy on LMIC islands could influence the food choices of the population [18, 19]. The children demonstrated awareness of healthy dietary choices but lacked consistent access to nutritious food, consistent with analyses that identify affordability and availability as persistent barriers in small island developing states [20]. These determinants reinforce the necessity of integrated approaches that address oral health alongside broader food system reforms [21, 22, 23].

Consistent with global oral health literature, the scarcity of dental professionals and the high out‐of‐pocket costs represent challenging barriers to effective preventive care [13, 24, 25]. Although attitudes towards oral health might influence the utilization of services, our study indicated that the absence of these services and high costs of existing services were the most significant barrier [25]. Issues with the availability and provision of dental services have been highlighted by previous research as a barrier to improving oral health [7, 9, 13, 26, 27]. The financial barrier and its association with poor oral health are also in keeping with research from high‐income countries like the United States which have shown that low‐income children's oral care is worse than that of middle‐ or high‐income children [4, 6, 14, 28]. Factors that contribute to this are the cost due to lack of dental insurance and difficulties finding a dentist [29, 30].   Although the existing governmental provisions are well known by officials, this knowledge is more limited at the population level. It might also be that indirect costs linked to accessing care limited parents’ abilities to utilize these services [31]. Additionally, our study showed a community movement that could potentially be utilized for the provision of care. Research studies in multiple health domains, including maternal and child health, have shown that community health workers and community groups can be significant drivers for improved health outcomes [32, 33]. Although the research studies are inconclusive, there appears to be a growing interest in exploring the roles of community health workers and community groups in improving oral health [32, 34, 35, 36]. Such interventions may be particularly viable in the island context of Cabo Verde.

From a policy perspective, our findings underscore the need for integrated food and health policies that go beyond educational campaigns. These include enhanced health promotion through community organizations and school‐based programs, increased availability of oral health tools and reduced access and consumption of sugary foods. Our findings suggest that schools and parents are already engaged in efforts to improve oral health knowledge and behaviors. However, without dental care providers, achieving a good population‐level oral health remains unlikely. Other island nations could provide a template for achieving improved oral health in such a constrained setting with a limited number of dentists and changing dietary patterns. For example, the Mali program in Tonga, a school‐based dental health promotion initiative, has led to a reduction of decayed missing and filled teeth [9, 18].

### 4.1 Limitations

Our study has some limitations which should be considered when considering our results. In keeping with qualitative approaches, we adopted a purposive sampling approach. However, the sample was not fully representative, with participants mainly drawn from two communities on Sal, and recruitment of parents proved particularly challenging. Our participants, consequently, did not represent all locations in Sal; rather, they were from Santa Maria and Espargos. There might be some different contextual challenges in other locations. Time constraints were a frequently cited reason for declining participation, which possibly restricted the breadth of perspectives. During the interviews and FDGs, information might have been lost in translation. Although a professional translator who was part of the team and fully aware of the pitfalls of translation and its effect on research results, nuances may have been lost in translation, and subtle shifts in meaning could have arisen depending on wording choices. Translated versions of the interview questions may also have influenced how participants framed their responses. Although we attempted to minimize these issues through continuous dialogue with the translator and iterative feedback during data collection, translation remains a potential source of bias.

## 5 Conclusion

Our findings indicate that although education and prevention programs are essential, addressing oral health challenges in Cabo Verde requires broader system‐level changes. This includes reviewing the impact of tourism, evolving dietary habits, and strengthening habits like daily toothbrushing practice using fluoride toothpaste, alongside improving access to affordable care.

## Funding

The authors have nothing to report.

## Author Contributions

Onaedo Ilozumba: Conceptualisation, formal analysis, methodology, writiting – original draft, writing – review & editing Marijke W. Visser: Methodology, investigation, project adminsttration, formal analysis, writiting – original draft, writing – review & editing J.J (Hans) de Soet: Conceptualization, resources, writing – reviewing & editing, supervision Catherine M.C Volgenant: Conceptualization, resources, visualization, writing – reviewing & editing, supervision.

## Ethics Statement

The study protocol was approved by the internal review board of ACTA (reference number 2018012). The study was also approved by the municipality of Sal.

## Consent

Informed consent was obtained from the participants, and, for children under 18 years of age, consent was additionally secured from their caregivers.

## Conflicts of Interest

The authors declare no conflicts of interest.

## Supporting information




**Supporting file 1**: puh270184‐sup‐0001‐Appendix1.docx

## Data Availability

Data will be provided upon reasonable request.
