# Supplementary material for: Exploring Oral Health Challenges and Barriers to Dental Care Among Children in Cabo Verde: A Qualitative Study
Source: Public Health Chall. 2026 Jan 13;5(1):e70184. doi: 10.1002/puh2.70184 (PMC12800389; doi:10.1002/puh2.70184)
Supplement: Supplementary file 1 — Supporting file 1: puh270184‐sup‐0001‐Appendix1.docx [file PUH2-5-e70184-s001.docx]

### Part 1. An overview of the definition of oral health

1. *What is your understanding of oral health?*
   1. *Rephrasing question: What does a healthy mouth mean?*
2. *What is needed to maintain good oral health according to you?*
   1. *How do you maintain good oral health?*
   2. *Rephrasing question: How do you take care of your mouth and teeth?*
   3. What are examples of practices you do to maintain good oral health, such as *teeth brushing, toothpick, sugarless diet?*
3. *What are risk factors for oral health according to you?*
   1. *What do think decreases oral health?*
   2. *Rephrasing question: What harms your mouth and teeth?*
   3. *Can you name examples of what harms the oral health, such as sugar, alcohol, and tobacco?*

### Part 2. The behavioral beliefs and attitude concerning oral health

1. *What do you think of oral health in general?*
   1. *Can you explain what kind of discomforts you had in your life regarding oral health?*
2. *What is the order of importance of the practices for good oral health?*
3. *What is the order of importance of the risk factors for oral health?*
4. *Can you explain to me why you have put them in this order?*

### Part 3. The normative beliefs and subjective norms concerning oral health

1. *What are the customary practices for good oral health?*
2. *What are the customary risk factors?*
3. *What do you think of these customs?*
4. *How do you think these customs can be influenced?*
5. *What needs improvement in the current oral health education?*

### Part 4. The control beliefs and perceived behavioral control concerning oral health

1. *What facilities are provided to you for oral health?*
2. *What facilities undermine your oral health?*
3. *What do you think about these provided and undermining facilities?*
4. *What do you think needs to be changed to improve these facilities?*
